# Correction: Guarneri et al. Diagnostic Performance of PIVKA-II in Italian Patients with Hepatocellular Carcinoma. *Cancers* 2025, *17*, 167

**DOI:** 10.3390/cancers17091526

**Published:** 2025-04-30

**Authors:** Valeria Guarneri, Elisabetta Loggi, Giuseppe Ramacieri, Carla Serra, Ranka Vukotic, Giovanni Vitale, Alessandra Scuteri, Carmela Cursaro, Marzia Margotti, Silvia Galli, Maria Caracausi, Lucia Brodosi, Filippo Gabrielli, Pietro Andreone

**Affiliations:** 1 Department of Medical and Surgical Sciences, University of Bologna, 40138 Bologna, Italy; valeria.guarneri2@unibo.it (V.G.); giuseppe.ramacieri2@unibo.it (G.R.); alessandrascuteri@hotmail.com (A.S.); 2 Operational Unit of Clinical Pathology, ASUR4, 63900 Fermo, Italy; bettaloggi@gmail.com; 3 Interventional, Diagnostic and Therapeutic Ultrasound Unit, IRCCS AOUBO, 40138 Bologna, Italy; carla.serra@aosp.bo.it; 4 Department of Emergency and Acceptance, General Medicine IV, University Hospital of Pisa, 56124 Pisa, Italy; ranka.vukotic@ao-pisa.toscana.it; 5 Internal Medicine Unit for the Treatment of Severe Organ Failure, IRCCS AOUBO, 40138 Bologna, Italy; giovanni.vitale@aosp.bo.it; 6 Department of Medical and Surgical, Maternal-Infantile and Adult Sciences, University of Modena and Reggio Emilia, 41126 Modena, Italy; carmela.cursaro2022@libero.it (C.C.); marzia.margotti2@unibo.it (M.M.); pietro.andreone@unimore.it (P.A.); 7 Microbiology Unit, IRCCS AOUBO, 40138 Bologna, Italy; silvia.galli@aosp.bo.it; 8 Unit of Histology, Embriology and Applied Biology, Department of Biomedical and Neuromotor Sciences, University of Bologna, 40126 Bologna, Italy; maria.caracausi2@unibo.it; 9 Clinical Nutrition and Metabolism Unit, IRCCS AOUBO, 40138 Bologna, Italy

**Text Correction**

There was an error in the Results section of the original publication [1]: “The original contributions presented in this study are included in the Supplementary Materials”. A correction has been made and this has been updated to “The dataset is available only upon request to the first author”.

There was an error in Section 4.1 of the original publication [1] “*4.1. What Does the Study Add to Current Knowledge?*”: “To further support this, we have included the dataset in the Supplementary Materials to encourage its use in future meta-analyses”. This sentence has been removed.

There was an error in the Supplementary Materials note included in the original publication [1]: “The following supporting information can be downloaded at: https://www.mdpi.com/article/10.3390/cancers17020167/s1, Dataset of subjects with Hepatocarcinoma (HCC) selected in this study”. This sentence has been removed.

There was an error in Section 3.5 of the original publication [1], “*3.5. Analysis of Diagnostic Threshold for PIVKA-II*”: “The area under curve (AUC) was found to be 0.816”. This has been changed to “The area under the curve (AUC) was found to be 0.817”.

There was an error in Section 3.6 of the original publication [1], “*3.6. Comparison of Diagnostic Thresholds for PIVKA-II and AFP*”: “The comparison of PIVKA-II and AFP as diagnostic tools for HCC revealed superior performance for PIVKA-II. Specifically, the AUC for AFP was 0.674 (95% CI 0.598–0.750, *p* < 0.0001), which was significantly lower than the AUC of PIVKA-II (0.815; 95% CI 0.754–0.876 *p* < 0.0001)”. This has been corrected to “The comparison of PIVKA-II and AFP as diagnostic tools for HCC revealed superior performance for PIVKA-II. Specifically, the AUC for AFP was 0.674 (95% CI 0.596–0.750, *p* < 0.001), which was significantly lower than the AUC of PIVKA-II (0.815; 95% CI 0.754–0.876 *p* < 0.001)”.

There was an error in the Data Availability Statement in the original publication [1]: “The dataset used during the current study is available from the corresponding author on reasonable request”. This has been corrected to “The dataset is available only upon request to the first author”.

**Remove Supplementary Materials**

There was an error in the Supplementary Materials in the original publication [1], the file has been removed.

**Error in Table**

In the original publication, there was a mistake in Table 1 as published. The corrected Table 1 appears below.

The authors apologize for any inconvenience caused and state that their scientific conclusions are unaffected. This correction was approved by the Academic Editor. The original publication has also been updated.

## Figures and Tables

**Table 1:** Baseline clinical and demography features of patients enrolled.

Variable	HCC *N* = 80	LC *N* = 111	CHC *N* = 111
Median Age (Years)	66 [48–85]	63 [20–83]	59 [28–84]
Male/female: N [%]	65/15 (81%/19%)	67/44 (60%/40%)	41/70 (37%/63%)
Liver Disease Etiology			
Hepatitis C infection	19 (23.75%)	46 (42%)	104 (94%)
Hepatitis B (±Delta Coinfection)	7 (8.75%)	11 (10%)	0 (0%)
Alcohol abuse	10 (12.5%)	5 (4%)	0 (0%)
NAFLD	4 (5%)	9 (8%)	0 (0%)
Cryptogenetic disease	2 (2.5%)	2 (2%)	0 (0%)
Multifactorial disease:			
Viral + Alcohol	2 (2.5%)	14 (13%)	1 (1%)
Metabolic + Alcohol	10 (12.5%)	5 (4%)	0 (0%)
Viral + Metabolic	3 (3.75%)	13 (12%)	6 (5%)
Metabolic + Autoimmune	1 (1.25%)	1 (1%)	0 (0%)
Mixed etiology *	23 (28.75%)	5 (4%)	0 (%)
CTP Score			
A	63 (78.75%)	102 (92%)	110 (99%)
B	17 (21.25%)	9 (8%)	1 (1%)
Meld Score	9 [3–16]	8 [1–22]	5 [0–9]
BCLC Score			
0	25 (31.25%)		
A	33 (41.25%)		
B	22 (27.5%)		

* More than three risk factors. HCC = hepatocellular carcinoma, LC = liver cirrhosis, CHC = chronic hepatitis C, CTP = Child–Turcotte–Pugh, N = number of subjects, NAFLD = non-alcoholic fatty liver disease, BCLC = Barcelona Clinic Liver Cancer. Categorical variables are reported as absolute number (% of total) while continuous variables are reported as median [min–max].
